# Contrast-Enhanced Ultrasound for Assessing Abdominal Conditions in Pregnancy

**DOI:** 10.3390/medicina56120675

**Published:** 2020-12-08

**Authors:** Thomas Geyer, Johannes Rübenthaler, Matthias F. Froelich, Laura Sabel, Constantin Marschner, Vincent Schwarze, Dirk-André Clevert

**Affiliations:** 1 Department of Radiology, University Hospital LMU, Marchioninistrasse 15, 81377 Munich, Germany; johannes.ruebenthaler@med.uni-muenchen.de (J.R.); lauranina.sabel@gmail.com (L.S.); constantin.marschner@med.uni-muenchen.de (C.M.); vincent.schwarze@med.uni-muenchen.de (V.S.); dirk.clevert@med.uni-muenchen.de (D.-A.C.); 2 Department of Radiology and Nuclear Medicine, University Medical Centre Mannheim, Theodor-Kutzer-Ufer 1-3, 68167 Mannheim, Germany; matthias.froelich@medma.uni-heidelberg.de

**Keywords:** CEUS, contrast-enhanced ultrasound, pregnancy, placental barrier, off-label use, fetus

## Abstract

*Background and objectives*: Native ultrasound is the most common imaging modality in obstetrics. The use of contrast-enhanced ultrasound (CEUS) during pregnancy has not been officially approved by leading societies for obstetrics and ultrasound. The present study aims to monitor the safety and diagnostic performance of CEUS for assessing abdominal issues in five pregnant women. *Materials and Methods*: Five pregnant patients who underwent a total of 11 CEUS examinations between June 2020 and October 2020 were included (mean age: 34 years; mean time of pregnancy: 21 weeks). All CEUS scans were interpreted by one experienced consultant radiologist (EFSUMB Level 3). *Results*: Upon contrast application, no maternal nor fetal adverse effects were observed. Moreover, no fetal contrast enhancement was observed in any patient. CEUS helped to diagnose renal angiomyolipoma, pyelonephritis, necrotic uterine fibroid, gallbladder polyp, and superior mesenteric vein thrombosis. *Conclusions*: In our study, off-label use of CEUS showed an excellent safety profile allowing the avoidance of ionizing radiation exposure as well as contrast agents in case of CT or use of gadolinium-based contrast agents in case of MRI. CEUS is a promising diagnostic instrument for facilitating clinical decision-making and improving the management of pregnant women.

## 1. Introduction

Ultrasound is the most frequently used imaging modality in obstetrics. The International Society of Ultrasound in Obstetrics and Gynecology (ISUOG) recommends a first-trimester fetal ultrasound scan to assess viability, number of fetuses, gestational age, amnionicity, and chorionicity [1]. Native ultrasound is widely available and has an excellent safety profile. Even though the application of ultrasound in pregnancy may lead to potential harmful effects by local tissue heating [2], no fetal nor maternal adverse effects of its use during pregnancy have been reported yet. Nevertheless, a variety of conditions in pregnant patients require more elaborate imaging modalities and the application of contrast agents [3,4].

In case of non-obstetric, life-threatening emergencies during pregnancy, benefits of directly accessible CT may outweigh potential fetal and maternal risks, including carcinogenic and teratogenic effects, due to ionizing radiation [5].

Performing MRI during pregnancy requires thorough evaluation. Compression of the inferior vena cava (IVC) during supine position of the pregnant patient may prompt arterial hypotension, especially at late gestational ages [6,7]. Left-lateral tilt position may prevent the compression of IVC [8]. Furthermore, potential fetal side effects of MRI such as tissue heating and acoustic damage were previously discussed [5,9]. MRI-induced hyperthermia, for example, may not be of high relevance as scanning times are kept to a minimum with body temperature usually not increasing by more than 0.5 °C [10]. Since there is not yet any definitive evidence for its safety, the application of gadolinium-based contrast agents (GBCAs) during pregnancy is still controversial [11]. According to a guidance statement by the American College of Radiology (ACR), intravenous application of GBCAs should be avoided in pregnant patients and only be used if absolutely necessary [12]. Previous publications showed that GBCAs cross the placental barrier and enter fetal circulation [13,14,15]. However, preclinical studies in nonhuman primates demonstrated that the gadolinium concentration in the fetoplacental circulation after maternal gadolinium injection is minimal compared to the concentration levels in the maternal circulation [16]. Although increased levels of the gadolinium concentration in the amniotic fluid after maternal injection during pregnancy were detected in rhesus macaques, only a minute fraction of gadolinium was found in juvenile tissue at follow-up [13]. Of note, gadolinium deposition in the central nervous system, especially in the basal ganglia were recently described. Unknown so far are the potentially induced long-term clinical side-effects [17,18]. An in vitro study showed that GBCAs can disrupt the interactions between thyroid hormones and their receptors and may therefore cause toxic adverse effects in the brain [19]. An animal study evaluating the effects of perinatal use of GBCAs showed that intravenous injection of GBCAs in pregnant mice leads to increased cerebral gadolinium deposition during postnatal development and is associated with motoric and neural disorders [20]. Due to the lack of substantial evidence of gadolinium’s safety in pregnancy, the American College of Radiology (ACR) Committee on Drugs and Contrast Media recommends the intravenous application of GBCAs only when “potential benefits justify the potential unknown risk of the fetus” [21].

First publications about CEUS in pregnant patients investigated the blood flow in women with monochorionic twin fetuses [22,23]. The first published studies already described the safe application of CEUS for evaluating invasive placenta percreta, cesarean scar pregnancy, and uteroplacental blood flow [24,25,26]. The availability of data about safe off-label use of CEUS during pregnancy is, to this day, still limited. Additional recent studies about the utility of CEUS for assessing non-obstetric conditions in pregnant women demonstrated promising results. The use of CEUS for diagnosing liver echinococcosis in a pregnant patient was described in one case report [27]. Moreover, recent publications demonstrated the safe use of CEUS for assessing hepatic lesions in six pregnant patients [28] and various abdominal conditions in five patients [29], respectively. However, up until now, the application of contrast-enhanced ultrasound (CEUS) during pregnancy has not been officially approved by leading societies for obstetrics and ultrasound due to concerns about potential related adverse effects [30]. Since only a limited amount of data using CEUS in obstetrics exists, further research needs to be done.

The present study aims to monitor the safety and the diagnostic performance of CEUS for assessing abdominal issues in pregnant patients.

## 2. Materials and Methods

The local institutional ethical committee of the institutional review board (Ethics Committee, Medical Faculty, Ludwig-Maximilians-University Munich; 17-087; date of approval: 14 March 2017) approved this retrospective single-center study. All contributing authors followed the ethical guidelines for publication in *Medicina*. Data used for our study were collected in compliance with the principles expressed in the Declaration of Helsinki/Edinburgh 2002.

All CEUS examinations were performed and interpreted by an experienced consultant radiologist (EFSUMB Level 3) using an up-to-date high-end ultrasound device (*Philips EPIQ 7*, transducer C5-1, Seattle, Washington, DC, USA) and appropriate CEUS protocols. Oral and written informed consent of all women included in the study was obtained before the examination. Potential maternal risks including allergic reactions, anaphylactic shock, dyspnea, arterial hypotension, bradycardia, dizziness, paresthesia, erythema, nausea, and vomiting as well as potentially unknown fetal complications up to miscarriage and fetal abnormalities all had been thoroughly explained. All scans included native B-Mode ultrasound, Color Doppler, and CEUS. Fetal cardiac activity and fetal movement were assessed by B-mode and Color Doppler before and after each contrast-enhanced examination. CEUS scans were performed using a second-generation blood pool contrast agent (*SonoVue^®^,* Bracco, Milan, Italy). A bolus of 1.5–2.4 mL of *SonoVue^®^* was injected intravenously, followed by 5–10 mL of sterile 0.9% sodium chloride solution. A low mechanical index (<0.2) was used for all examinations to avoid early destruction of microbubbles. Lesions were assessed in the early arterial phase (10–45 s after intravenous contrast injection) and in the late phase (120–150 s after intravenous contrast injection). No maternal or fetal adverse side effects upon intravenous application of *SonoVue^®^* were registered. Cine-loops were acquired upon application of the contrast agent and archived in our institutional picture archiving and communication system (PACS). Image quality was sufficient in all examinations.

We included a total of five pregnant women in our single-center study who underwent a total of 11 CEUS examinations.

## 3. Results

All 11 CEUS examinations were performed between June 2020 and October 2020. The mean age of the five included pregnant patients was 34 years (range: 30–37 years), the mean time of pregnancy at the time of CEUS examination was 21 weeks (range: 14–31 weeks) (Table 1).

**Figure 1:**
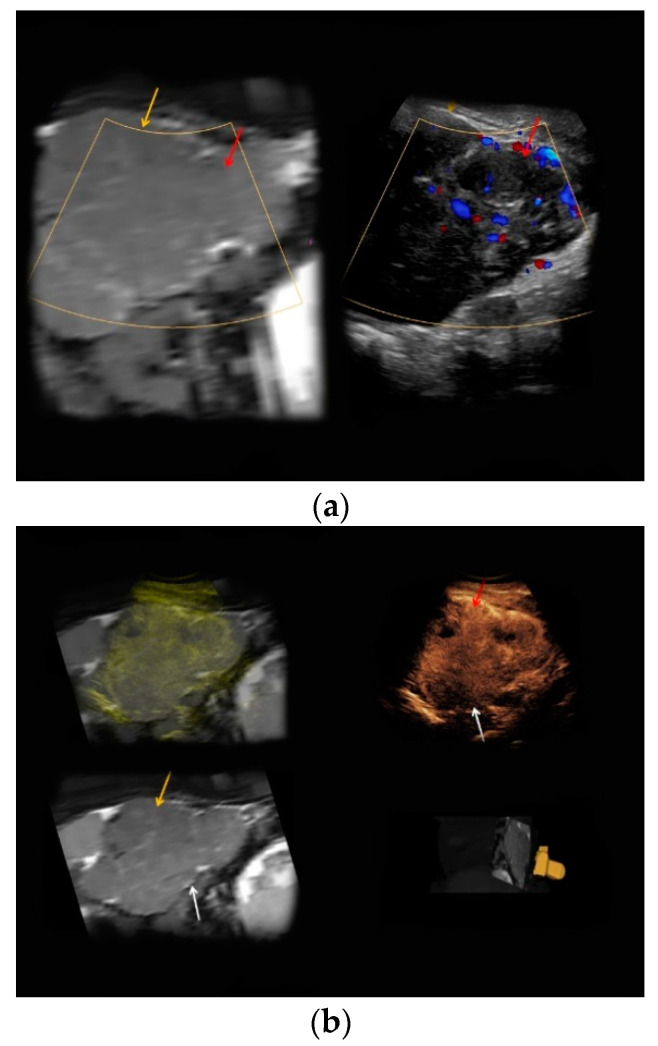
Renal angiomyolipoma, 25 weeks of pregnancy, real-time MRI-/CEUS-Fusion Imaging. (**a**) T2-hypointense tumor (yellow arrow) with 14 cm in diameter of the left kidney in unenhanced MRI (left, red arrow—renal parenchyma), T2-weighed image, axial reformation; displayed in a side-by-side manner with corresponding Doppler mode, which revealed no lesional hypervascularization (right, yellow arrow) compared to adjacent renal parenchyma (right, red arrow). (**b**) Wash-out during the late phase of the tumor was detected during CEUS (upper right, white arrow), no wash-out of the renal parenchyma (red arrow); correlate of unenhanced MRI (lower left, yellow arrow—renal parenchyma, white arrow—renal tumor); fused correlates of CEUS and MRI (upper left); real-time 3D navigation of the CEUS-/MRI-Image Fusion (lower right).

A 30-year-old woman at 27 weeks of pregnancy was referred to our department with an unclear renal tumor incidentally detected by her obstetrician. Using B-mode ultrasound, a hypoechoic lesion of 14 cm in diameter of the left kidney was registered. Upon intravenous application of *SonoVue^®^*, the lesion showed rapid contrast enhancement and slight wash-out during the late phase (Figure 1b, upper right). Previously performed, unenhanced magnetic resonance imaging (MRI) could not rule out malignancy (Figure 1a, left). After evaluation of the case within an interdisciplinary tumor conference, a biopsy of the suspicious renal lesion was taken. Histopathological analysis revealed a lipid-poor angiomyolipoma. Therefore, a watch-and-wait approach until childbirth was conducted instead of immediate surgery. Follow-up CEUS examinations were performed at 28, 29, 30, and 31 weeks of pregnancy to rule out tumor growth and infiltration of blood vessels, thus preventing potentially life-threatening complications such as internal bleeding. However, the renal angiomyolipoma remained unchanged during follow-up, and the woman gave birth to a healthy neonate at 38 weeks of pregnancy by vaginal delivery.

A 37-year-old pregnant woman at 21 gestational weeks presented at our institutional Emergency Department due to severe left flank pain, chills, and fever. Laboratory testing revealed markedly elevated levels of C-reactive protein (CRP) and white blood cell (WBC) count. Urinalysis indicated a urinary tract infection. In B-mode ultrasound and Doppler mode, no renal abnormalities could be registered. In CEUS, the renal parenchyma showed homogeneous contrast enhancement, with no renal abscess found. Subsequent unenhanced MRI confirmed these findings, thus indicating acute pyelonephritis. Calculated empiric antibiotic therapy was initiated with cefuroxime and switched to meropenem after two days due to a further increase in serum inflammatory markers and persistent fever. Ten days after therapy initiation, the inflammatory parameters returned to normal levels with the patient being discharged from the hospital without persistent symptoms.

**Figure 2:**
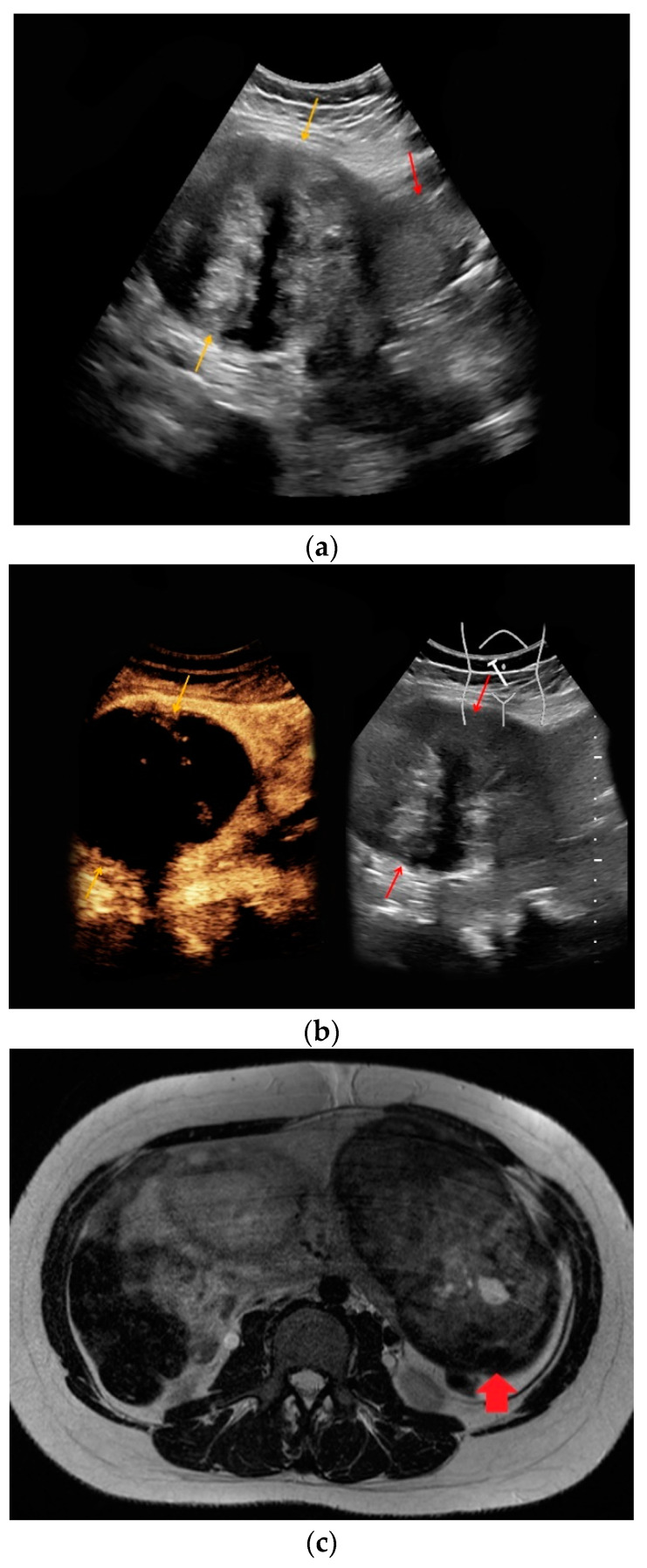
Necrotic uterine fibroid, 25 weeks of pregnancy. (**a**) Inhomogeneous, hypo-/hyperechoic lesion of the uterus with 15 cm in diameter in B-mode (yellow arrows; red arrow: uterus). (**b**) Only a slightly marginal contrast enhancement was detected in CEUS (yellow arrow). (**c**) In adjunct MRI, a predominantly T2-hypointense, inhomogeneous lesion of the left uterine wall was visualized (red arrowhead).

Due to an incidentally detected unclear uterine tumor, a 34-year-old patient at 25 weeks of gestational age was referred to our department by her caring obstetrician. The woman suffered lower abdominal pain with blood tests having revealed elevated markers of inflammation. Contrast-enhanced ultrasound was performed for the definitive characterization of the uterine lesion. A 15 cm inhomogeneous tumor at the left uterine wall could be visualized in B-mode ultrasound (Figure 2a). The lesion showed no hypervascularization in Doppler mode and only a slight contrast enhancement in CEUS (Figure 2b). The morphological findings led to the diagnosis of necrotic uterine fibroid confirmed by MRI (Figure 2c). Under calculated antibiotic therapy, the serum inflammatory markers returned to normal levels and the patient was discharged to ambulatory care after a hospitalization period of five days.

**Figure 3:**
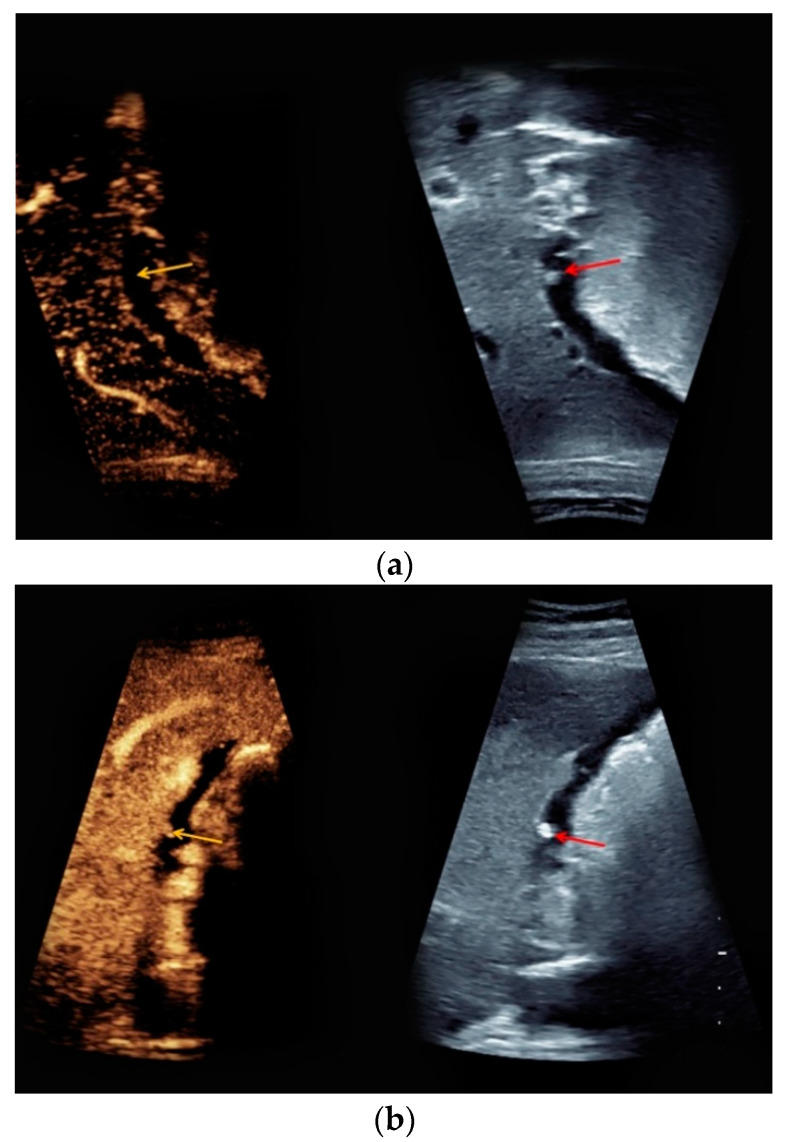
Gallbladder polyp, 17 weeks of pregnancy. (**a**) Upon intravenous contrast application, the mural lesion of the gallbladder displaced in a side-by-side-manner with corresponding B-mode (right, red arrow), showing no contrast enhancement in the early phase (left, yellow arrow), (right, lesion—red arrow). (**b**) In the late phase, slight homogeneous contrast uptake of the polyp was visualized (yellow arrow), displayed in a side-by-side-manner with corresponding B-mode (right, lesion—red arrow).

A 33-year-old woman presented for further characterization of an incidentally detected gallbladder lesion. The gestational age at the time of CEUS was 17 weeks. Native ultrasound showed a hyperechoic mural lesion of the gallbladder with a diameter of 0.6 cm and no signs of hypervascularization. Upon contrast application, the lesion showed no contrast enhancement in the early phase (Figure 3a) but rather a slight, homogeneous contrast enhancement in the late phase (Figure 3b). Findings from CEUS led to the diagnosis of a gallbladder polyp. Since the patient was asymptomatic with no signs of malignancy registered, no further treatment before childbirth was required.

**Figure 4:**
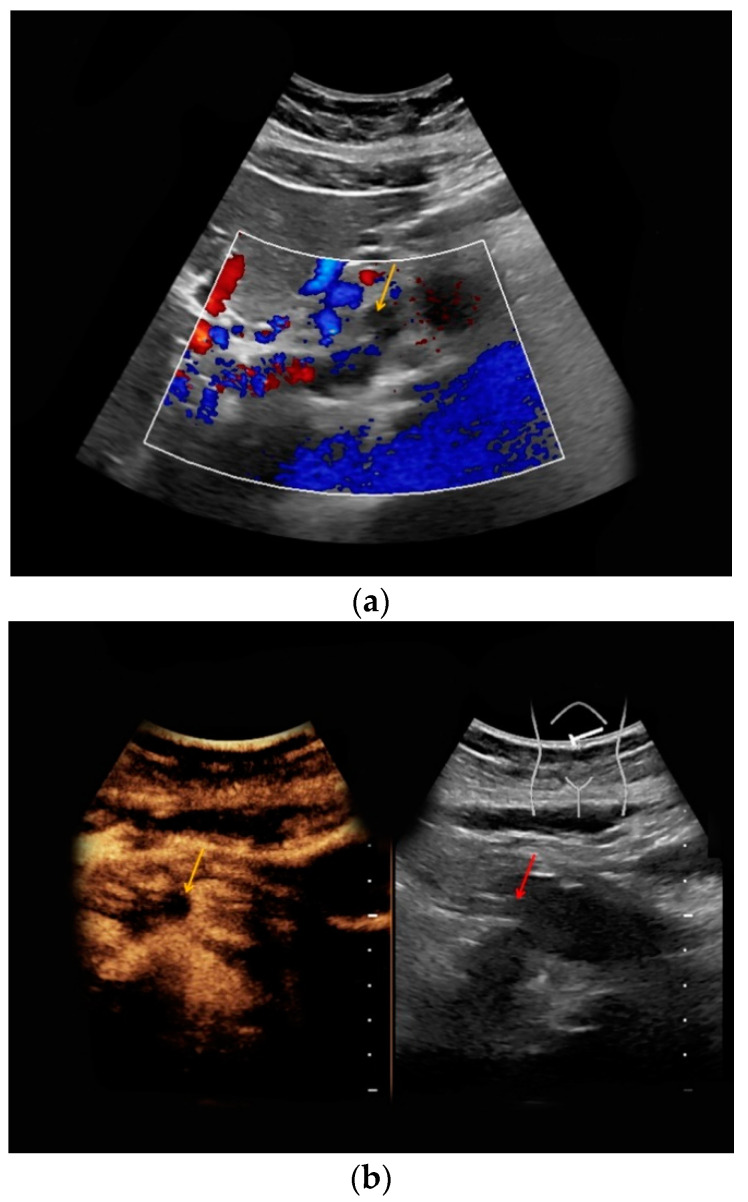
Superior mesenteric vein thrombosis, 14 weeks of pregnancy. (**a**) No internal Doppler signal in the superior mesenteric vein in Doppler mode (yellow arrow). (**b**) Upon intravenous contrast application, only very few microbubbles were marginally detected in the superior mesenteric vein due to the thrombosis (yellow arrow).

A 34-year-old pregnant woman was referred to our department. Twenty-one years before, the patient had undergone mesenteric-left portal vein bypass surgery during early adolescence due to congenital extrahepatic portal vein thrombosis. Furthermore, resection of the rectum, the sigmoid colon, and the cecum with the appendix was performed twelve years ago due to rectal varices and recurring gastrointestinal bleeding. During a previous pregnancy two years before, a superior mesenteric vein thrombosis was detected; hence, oral anticoagulation therapy being initiated. The woman gave birth to a healthy neonate by cesarean section, with follow-up ultrasound examinations showing residual thrombosis of the superior mesenteric vein (Figure 4). Oral anticoagulation was continued, and the thrombosis remained unchanged in follow-up CEUS examinations at 17, 22, and 24 weeks of pregnancy as well as at an additional MRI at 22 weeks of pregnancy.

**Figure 5:**
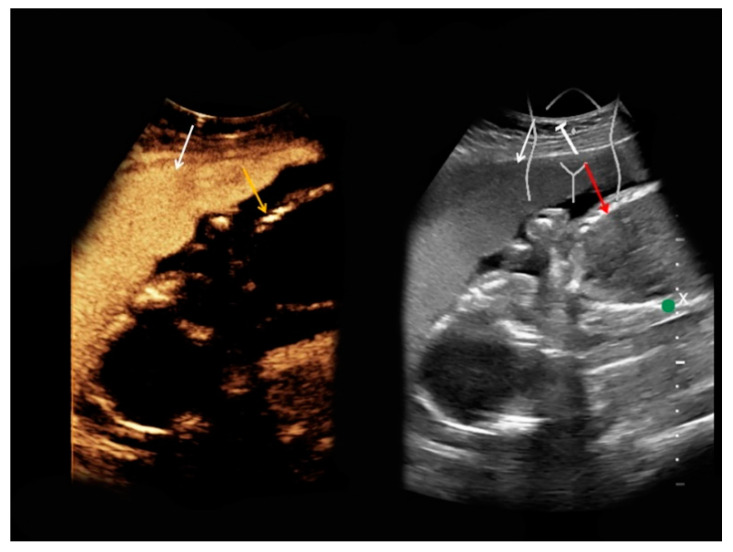
Intrauterine fetus at 25 weeks of pregnancy. Upon intravenous contrast application, beside a broad placental contrast enhancement (left, white arrow), no fetal contrast uptake was registered (left, yellow arrow); this is displayed in a side-by-side manner with corresponding B-mode (right, placenta—white arrow, fetus—red arrow).

In none of the five pregnant women included in our study was fetal contrast uptake detected during CEUS (Figure 5).

Without a doubt, in this study, the diagnostic work-up including diagnostic imaging would have been different, if the included patients had not been pregnant. In patient #1, contrast-enhanced cross-sectional imaging or CEUS would have been conducted to assess renal angiomyolipoma. In patient #2, contrast-enhanced CT or MRI would have been used for evaluating concomitant renal abscess in pyelonephritis. In patient #3, cross-enhanced cross-sectional imaging would have been performed in the case of a necrotic uterine fibroid. In patient #4, the gallbladder polyp would have been analyzed by means of a contrast-enhanced MRI. In patient #5, a superior mesenteric vein thrombosis would have been investigated by contrast-enhanced MRI.

## 4. Discussion

Native ultrasound is the most common imaging modality in obstetrics. Its use is safe during all stages of pregnancy and is recommended by the leading societies for obstetrics and ultrasound [30]. Up to date, there have been no documented fetal nor maternal adverse effects for native B-mode ultrasound or Color Doppler imaging. Conventional ultrasound may be safely applied during pregnancy when appropriately conducted and medically indicated [31]. Nevertheless, its diagnostic value has several limitations, including maternal obesity, fetal positioning, and difficulties in penetrating air, gas, or bone [11]. Hence, a variety of obstetric and non-obstetric conditions necessitate cross-sectional imaging and intravenous application of contrast agents for definitive characterization [3,4].

Contrast-enhanced ultrasound is a non-ionizing, widely available, and cost-effective imaging modality with an excellent safety profile [32,33]. The rate of serious adverse effects to ultrasound contrast agents was assessed in clinical studies with large patient cohorts, with an incidence of 0.0086% [32]. In early publications, the utility of CEUS for visualizing interfetal transfusion in monochorionic twin pregnancy was described in a total of 15 patients [22,23]. In an animal study, CEUS helped to visualize and quantify placental perfusion in pregnant rats, with no microbubbles being detected within the fetal structures and the umbilical vein, thus indicating that ultrasound contrast agents do not cross the placenta [34]. Besides that, a preclinical study could show that *SonoVue*^®^ does not increase the permeability of the placental barrier to macromolecules in a rat model [35]. Furthermore, no microbubbles could be registered in fetuses within this study. In a preclinical study investigating pregnant dogs, CEUS allowed for a safe evaluation of fetal and maternal blood flow without the occurrence of any adverse side effects or fetal contrast enhancement [36]. In 2016, a study investigating cesarean scar pregnancy in 92 patients showed a significantly higher diagnostic accuracy of CEUS compared to native ultrasound [25]. These results were confirmed by more recent studies of 30 and 485 cases of suspected cesarean scar pregnancy [37,38]. However, these studies only included women during the first trimester of pregnancy that did not plan to maintain pregnancy. One study investigating the plugging of spiral arteries in the placenta in 34 first trimester pregnant women reported no adverse effects after the use of CEUS [39]. Recently, a retrospective evaluation of the use of CEUS for assessing hepatic lesions in six pregnant women showed that CEUS reliably helped to differentiate malignant from benign liver lesions, including focal nodular hyperplasia, hepatic hemangioma, echinococcosis, hepatic arteriovenous malformation, and liver metastases in a rectal cancer patient [28]. Contrast-enhanced ultrasound allowed for safe differentiation between these lesions, while avoiding the use of GBCAs and exposure to ionizing radiation. No adverse fetal or maternal adverse effects upon application of the contrast agent occurred in any patient, and no contrast-enhancement in the fetal compartments was registered. A further recent study showed the safe application of CEUS for assessing a variety of non-obstetric conditions in five pregnant patients, including hepatic abscess and hemangioma, a desmoid tumor in the abdominal wall, and a Bosniak type 1 renal cyst. Contrast-enhanced ultrasound also helped to rule out active abdominal bleeding in a trauma patient and was used for guiding the placement of drainage into a hepatic abscess [29].

Our findings are in line with the results of previous studies. Fetal contrast uptake was not registered in any of the five pregnant women included in our study, indicating that microbubbles do not cross the placental barrier. No fetal nor maternal adverse effects upon administration of *SonoVue^®^* were observed. In patient #1, CEUS allowed for characterizing the dynamic contrast enhancement of an unclear lesion in the left kidney. Therefore, a subsequent MRI could be performed without using GBCA. Since differentiating renal angiomyolipoma from renal cell carcinoma can be difficult due to varying and overlapping imaging characteristics, the use of CEUS substantially contributed to an adequate diagnosis in conjunction with native MRI in patient #1 [40]. After renal angiomyolipoma was histopathologically confirmed, follow-up CEUS examinations allowed for continuing regular obstetric monitoring and avoiding immediate surgical tumor resection during pregnancy. In patients #2 and #3, CEUS helped to diagnose pyelonephritis and necrotic uterine fibroma, respectively, and excluded complications such as the presence of an abscess, which would have shown central necrosis and peripheral rim enhancement requiring further invasive treatment [41]. The necrosis of the uterine fibroid could be safely diagnosed by showing typical imaging characteristics with a lack of contrast enhancement [42]. By assessing contrast enhancement dynamics of a small gallbladder lesion in patient #4, CEUS led to the diagnosis of a gallbladder polyp by detecting typical homogeneous late contrast enhancement ruling out malignancy [43]. By this means, additional cross-sectional imaging associated with ionizing radiation or GBCA could be avoided. In patient #5, CEUS was used for monitoring a superior mesenteric vein thrombosis, thus preventing regular contrast-enhanced CT or MRI examinations. During CEUS examination, a typical lack of microbubble detection within the superior mesenteric vein helped to diagnose the thrombosis in this patient [44]. Applying CEUS crucially contributed to the diagnostic work-up and therapy management in the presented five pregnant patients, thereby avoiding additional contrast-enhanced CT or MRI.

Our results underline the safety and the potential diagnostic value of CEUS for assessing both obstetric and non-obstetric conditions during pregnancy. CEUS is a promising imaging modality that might allow for facilitating clinical decision-making and improving the management of pregnant patients in the future.

Limitations of this single-center study comprise its limited number of patients with only five pregnant women, the heterogeneity of the described abdominal conditions, the short period time for assessing potential adverse effects, and the retrospective study setting. Prospective clinical trials with larger patient cohorts and more extensive patient monitoring (e.g., cardiotocography) are needed to further evaluate the safety and the potential diagnostic value of using CEUS during pregnancy. 

## Figures and Tables

**Table 1:** Morphological appearance of abdominal conditions using CEUS and MRI in five pregnant patients.

Patient	Age (years)	Pregnancy (weeks)	Condition	Contrast-Enhanced Ultrasound (CEUS)	Magnetic Resonance Imaging (MRI)
#1	30	27	Renal angiomyolipoma	B-mode: hypoechoic, 14 cm Doppler: no hypervascularization CEUS: rapid early contrast enhancement, slight late wash-out	T1-hypointense, T2-hypointense, restricted diffusion
#2	37	21	Pyelonephritis, ruled out the presence of an abscess	B-mode: no abnormalities Doppler: no abnormalities CEUS: homogeneous contrast enhancement, no abscess	homogeneous, restricted diffusion, perirenal fluid collections
#3	34	25	Necrotic uterine fibroid	B-mode: inhomogeneous, hypo-/hyperechoic, 15 cm Doppler: no hypervascularization CEUS: slight contrast enhancement	inhomogeneous, predominantly T1-hypointense, T2-hypointense
#4	33	17	Gallbladder polyp	B-mode: hyperechoic, 0.6 cm Doppler: no hypervascularization CEUS: homogeneous, late contrast enhancement	-
#5	34	14	Superior mesenteric vein thrombosis	B-mode: no abnormalities Doppler: no Doppler signal in the superior mesenteric vein CEUS: only slightly marginal microbubble distribution in the superior mesenteric vein	small superior mesenteric vein thrombosis
